# Book Review

**Published:** 1931

**Authors:** 


### Reviews of Books

**Applied Physiology.** By Samson Wright, M.D., M.R.C.P. Fourth Edition. Pp. xxviii., 552. London: Oxford University Press. 1931. Price 18s.—The rapidity with which fresh editions of this work appear is ample testimony to its well-deserved popularity. In this, the fourth edition, the general format of the whole has remained, but it has been thoroughly revised and much new information added. By the simultaneous deletion of obsolete matter this addition has been accomplished without any increase in the size of the book. The fresh work thus incorporated includes the recent studies on the physiology of the sinus caroticus, a discussion on the various theories of biological oxidation, and the clinical features of hyperparathyroidism and hyperinsulinism, to mention only a few. The author thus maintains his intentions of illustrating the principles of physiology by reference to clinical observations on the human subject, and the whole work has been brought completely up to date from the point of view of both pure and applied physiology. This book is almost indispensable, not only to the physiology student, but also to the clinician anxious to keep abreast with recent advances in physiology as they affect clinical medicine.

**Ideal Marriage.** Pp. 323. Price 25s. **Sex Hostility in Marriage.** Pp. 296. Price 17s. **Fertility and Sterility in Marriage.** Pp. 448. Price 25s. A trilogy by T. H. van de Velde, M.D. (Haarlem). Illustrated. London: William Heinemann Ltd. 1931.—The author maintains that medical men in general stand much in need of instruction in the psychology and technique of married life. His first volume is a manual of practical erotics that certainly leaves nothing unsaid. Experienced lovers will admit the thoroughness and the insight displayed, but have probably nothing to learn here; for others detailed text-book instruction is not what is necessary. The psychological aspects are emphasized throughout, especially the importance of equal and reciprocal satisfaction. The second volume deals rather with the psychology of married life as a whole than of its more intense and intimate moments, though these are not left in the background. Considerable space is devoted to "Choice of a partner." Here again the accuracy of the author's observations might be easier to defend than the practical value of his advice.

The third volume is devoted to the much-discussed subject of birth control; factors promoting fertility receive consideration as well as procedure available for modifying them. Contraception is dealt with in some detail, ethics and psychology being again in the forefront; apart from operative measures the author has no suggestions for avoiding the real disadvantages of well-known methods. The trilogy as a whole deals with an aspect of normal human life that is of supreme importance to nearly every man and woman, and about which instructed opinion has hitherto been little accessible. The author is to be congratulated on the straightforward but sympathetic tone maintained throughout.

**Practical Morbid Histology.** By R. DONALDSON, M.D., F.R.C.S.E. Second Edition. Pp. ix., 487. 214 illustrations. London: William Heinemann Ltd. 1931. Price 42s.—This is a welcome and much-improved second edition of Professor Donaldson's excellent handbook, which has been thoroughly revised and rendered more complete by the addition of several new chapters. In the previous edition illustrations were purposely omitted; the value of the present one is enhanced beyond measure by the inclusion of over two hundred original drawings. In a book of this kind, which is intended for the use of students, good illustrations are indispensable and cannot be adequately replaced by the most lucid of verbal descriptions; the author is therefore to be congratulated on deciding—although apparently with some reluctance, judging from his prefatory note—for their inclusion in the present volume. The chapters are well arranged in the orthodox manner, and the subject-matter has been carefully chosen. Little space has been devoted to theoretical considerations, for which the student is referred to the larger standard text-books. Professor Donaldson's purpose is to provide a useful practical companion volume to these standard works, and in this he has succeeded admirably. The illustrations were drawn specially for this edition by the late Richard Muir, and, needless to say, are uniformly excellent, losing none of their value for being at times somewhat diagrammatic. The book should prove extremely useful to students during their professional course, as well as to those sitting for higher examinations.

**A Manual of General Medical Practice.** By W. STANLEY SYKES, M.B., M.R.C.S. Second Edition. Pp. xi., 213. London: H. K. Lewis & Co. Ltd. 1931. Price 7s. 6d.—This is a well-written book, and deals with the subject of general medical practice in a very clear and useful manner.

It is highly recommended to all those recently qualified, and should be very helpful to them in particular. The object of the book, as stated in the preface to the first edition, is very well carried out. As each chapter is complete in itself as dealing with the varied aspects of general practice work and all are good it is difficult to single out one from the other, but perhaps those on "The Diseases of General Practice," "Diagnosis," "Prognosis," and "Treatment" will be found most useful, while that on National Health Insurance work is very clear.

**The Cardiac Cycle.** By HARRINGTON SAINSBURY, O.B.E., M.D., F.R.C.P. Pp. 79. Bristol: John Wright & Sons Ltd. 1931. Price 5s.—The argument proposed in this essay is that the pulse wave is set in motion by the impact of the blood driven by the pressure of the ventricular systole against the under side of the aortic valves before these open; that the vibrations which this initiates are transmitted to the column of blood in the aorta and its branches; and that the wave thus propagated, by "milking" the lymph along the perivascular spaces is largely responsible for maintaining the lymphatic circulation. We trust that these views will receive the respect that is their author's due, as well as the criticism that his prefatory note invites.

**Clinical Notes on Disorders of Childhood.** By D. W. WINNICOTT, M.R.C.P. Pp. viii., 216. London: William Heinemann Ltd. 1931. Price 10s. 6d.—This book is one of the "Practitioner's Aid Series," and deserves the attention of every general practitioner. The author has selected a remarkable series of case-reports accompanied by a running commentary on such important subjects as the rheumatic heart, the significance of "growing pains," anxiety states, enuresis and convulsions. His enthusiasm makes the reader feel that it is, as Noël Coward would put it, an exciting thing to be a general practitioner, "who must be the pioneer in the study of minor disorders." Much of the family doctor's work is a "specialized form of friendship." The author stresses the connection between the development of symptoms with difficulties in the emotional development of the child. Not every reader will agree with the doctrines of therapeutic, even psychotherapeutic nihilism put forward, but everyone will be stimulated by Dr. Winnicott's fresh approach to matters of the greatest importance to the general practitioner.

**Radiology in Relation to Medical Jurisprudence.** By S. GILBERT SCOTT, M.R.C.S. Pp. x., 65. 24 plates. London: Cassell & Co. Ltd. 1931. Price 7s. 6d.—This little handbook adequately fulfils his aims as set out by the author in the preface. It describes many of those skeletal variations and other conditions which are liable to make accurate diagnosis difficult, and which often arise as problems to be decided in connection with claims for compensation in the Law Courts. The book is designed both for the medical man who uses X-rays for diagnosis, to help him to avoid making mistakes and to present radiological evidence efficiently in the witness box, and also for the legal profession, to help it to understand the properties of X-rays and their use and limitations in diagnosis. It consists of four chapters, covering in all some 60 pages, together with twenty-four excellently-reproduced radiographs. One might question the correctness of the author's views on one or two points, *e.g.* his notes on Kümmel's disease (p. 33), his terminology in the titles of Plates 9 and 10, and his suggestion that a hæmatoma will calcify in six days to the extent shown in Plate 15. But on the whole the handbook is well done, and is filled with information condensed in a form which should prove valuable to many who have not the time to familiarize themselves with the many normal variations and slight evidences of early pathology described in more detailed works.

**Fundamental Principles of Ray Therapy.** By W. BEAUMONT, M.R.C.S. Pp. viii., 124. 37 illustrations. London: H. K. Lewis and Co. Ltd. 1931. Price 6s. An elementary text-book should be clear and strictly accurate. This little book, which is certainly very elementary but neither clear nor accurate, treats ray therapy (we are told in the preface) in an unusual way. The number of errors in it is simply appalling. Need more be said about it ?

**The Epidemiology and Control of Malaria in Palestine.** By I. J. KLIGLER. Pp. xv., 240. Illustrated. Chicago: The University of Chicago Press. 1931. Price 22s. 6d.—This volume describes the control of malaria as carried out in certain districts of Palestine in the last nine years, and is of more than local interest, because it shows how the scientific study and control of the local mosquito carriers may be applied to other countries. Palestine has suffered terribly from the disease. Entire towns and villages have been wiped out by it, and the early Jewish colonies were planted in what turned out to be some of the worst districts. The malaria has certain peculiarities : the breeding season of the anopheles is remarkably prolonged, and there are two malarial special seasons in the year, viz. May to August and October to December. Complete hibernation does not exist. Even in January a few mosquitoes previously infected do enter houses carrying sporozoites, and cause new attacks. Again, though there are eight kinds of mosquitoes present, only three or four are of importance, and Kligler rightly lays stress on the folly and waste of attacking those which rarely infect man. Indeed, since this book appeared Swellingrebel has on the same principle changed the whole method of control in South Africa, showing that only two species there are important, and great sums have been wasted in drainage works for want of knowledge as to the true vectors. *A. Elutus* does most of the damage in Palestine, and breeds in stagnant or sluggish waters, while *M. Sergenti* and *M. Superpictus* in swift streams in the summer are also serious. In the salt water of a few marshes and around the Dead Sea we get *M. Multicolor*, and finally *A. Bifurcatus*, having developed a bad habit of breeding in house cisterns in towns, is much more harmful here than it is in Italy, where it leads an outdoor life. Our author, too, has the credit of proving that while the limit of direct flight is one and a half miles here and elsewhere, the actual range of dispersion of infected insects may be two and a quarter, and just before winter as much as five or seven miles. This, of course, is a very important matter. His method of control was, after a careful survey of the population and of the mosquitoes on the spot and for a mile or two around, (1) to drain stagnant pools and to clear away weeds from the water-courses, (2) to destroy larvæ by spraying every seven or ten days with Paris green or a crude oil mixture, and (3) to organize hunts for adult mosquitoes in houses and stables. The results were first rate, except in three districts where huge marshes could not be treated. It is remarkable that he got no success when he tried careful quinine administration to an entire population. This is a marked contrast to the good results reported in many countries, as collected by Sir Leonard Rogers (*Recent Advances in Tropical Medicine*). Kligler was himself much surprised at the failure, and though he allows that blackwater fever vanished and that the loss of working time was greatly reduced where quinine was given, he is quite certain that the incidence of the disease was not lessened. Certainly the difference is not explained by arguments that quinine is not a prophylactic though invaluable in treatment.

We can warmly recommend the book as a very able and painstaking contribution to the study of malaria. Every page shows the author's infinite capacity for taking trouble, every little detail is illustrated by elaborate maps, charts and tables of statistics. We may add that it is brightened by many charming little photographs of marsh scenes.

**Demonstrations of Physical Signs in Clinical Surgery.** By HAMILTON BAILEY, F.R.C.S. Third Edition. Pp. xx., 277. Illustrated. Bristol: John Wright & Sons Ltd. 1931. Price 21s.—We are not at all surprised that a third edition of this most excellent book has been called for in four years. Students, doctors and professional surgeons will all do well to read it. To master its contents is to go far towards becoming a good surgical diagnostician, and even to glance over its 318 illustrations, well chosen and well printed, is a quick and easy means of improving one's education. We are glad to see that the author calls attention to the value of trans-illumination in the diagnosis of swellings in the breast. There are a few misprints, *e.g.* in the title of Fig. 164.

**A Pocket Atlas and Text-Book of the Fundus Oculi.** By G. LINDSAY JOHNSON, M.D., F.R.C.S. Second Edition. Pp. ix., 215. Illustrated. London: Adlard & Son Ltd. 1931. Price 12s. 6d.—The twenty-seven beautiful plates of the fundus oculi, giving fifty-four illustrations of diseases of the fundus, are in every way excellent, and as each is combined with a very clear footnote, this book should prove a very useful guide to ocular conditions for the student and busy practitioner. These plates have an introduction in the letter-press, which, although not strictly dealing with the fundus, gives a very clear discussion of cataract, glaucoma, etc., and a concise description of methods of examination of the fundus and difficulties met with in certain cases.

**Warwick and Tunstall's "First Aid" to the Injured and Sick.** Thirteenth Edition. By F. C. NICHOLS, M.C., M.B., Ch.B. Pp. xii., 276. Illustrated. Bristol: John Wright & Sons Ltd. 1931. Price 2s. 6d.—Dr. Nichols is to be congratulated on the thirteenth edition of this well-known handbook on first aid. This book, which is intended for the advanced ambulance worker, amplifies the information given in the authorized text-book of the St. John Ambulance Association, and provides that additional instruction which will be so useful to those who have to assist in the training of junior first-aid students. The many illustrations are well reproduced, and the tabulation of treatment should be of value. The new chapter on "Gas Warfare" seems somewhat pessimistic, but will be of interest to those serving in territorial medical units, while of the other additions the chapter on "Competition Work" will be appreciated by the many men and women who take part in these competitions. One can confidently recommend this book to all those ambulance students who wish to pursue the subject beyond the elementary stage.

**Gould's Medical Dictionary.** Third Edition. By George M. Gould, A.M., M.D. Edited by R. J. E. Scott, M.A., B.C.L., M.D. Pp. xvi., 1,538. Illustrated. London: H. K. Lewis & Co. Ltd. 1931. Price 30s.—The third edition of this dictionary is well up to the high standard of previous issues. In addition to many thousands of words described, there are numerous useful tables of bacteria, operations, tests, etc. The editor calls attention to the simplification of orthography that is one of his principal aims, but he is inconsistent in the matter, frequently and apparently capriciously retaining the older spelling. Apart from this we have no criticism to offer, but the highest praise for the excellent production of this standard work of reference.

